# Geodiamolide A, a Marine Sponge Depsipeptide, Halts Proliferation and Triggers Cell Death in Squamous Cell Carcinoma (A431, NMSC) In Vitro

**DOI:** 10.3390/ijms27031293

**Published:** 2026-01-28

**Authors:** Marisa Rangel, Alicia S. Ombredane, Ricardo B. Azevedo, Wagner Fontes, Graziella A. Joanitti, Mariana S. Castro

**Affiliations:** 1 Laboratory of Immunopathology, Butantan Institute, Av. Vital Brasil, 1500, Butantã, São Paulo 05503-900, SP, Brazil; marisarangel2112@gmail.com; 2 Laboratory of Toxinology, Department of Physiological Sciences, Institute of Biology, University of Brasília, Brasília 70910-900, DF, Brazil; 3 Laboratory of Nanobiotechnology, Department of Genetics and Morphology, Institute of Biology, University of Brasília, Brasília 70910-900, DF, Brazil; aliciaombredane@gmail.com (A.S.O.); razevedo@unb.br (R.B.A.); bygra1@gmail.com (G.A.J.); 4 Laboratory of Bioactive Compounds and Nanobiotechnology, Faculty of Ceilândia, University of Brasília, Brasília 72220-275, DF, Brazil; 5 Laboratory of Protein Chemistry and Biochemistry, Department of Cell Biology, Institute of Biology, University of Brasília, Brasília 70910-900, DF, Brazil; wagnerf@unb.br

**Keywords:** human squamous cell carcinoma, A431 cell lineage, depsipeptide, geodiamolide, cell death

## Abstract

Geodiamolides are depsipeptides previously isolated from marine sponges that are able to disrupt cytoskeleton microfilaments, inhibit cell migration and invasion, and reverse the malignant phenotype of human breast cancer cell lines to polarized spheroid-like structures. Such cytotoxicity to different cellular targets in breast cancer cells suggests that these molecules might also act in other cancer types such as non-melanoma skin cancer (NMSC), one of the cancer types with high incidence worldwide. Thus, the goal of this work was to study the effects of the marine sponge depsipeptides Geodiamolide A and H (Geo A and Geo H) in human squamous cell carcinoma (A431, NMSC) in order to investigate their effects on cell proliferation and cell death. While no significant statistical difference was observed after Geo H treatment, an expressive dose-dependent reduction in A431 cell viability (IC_50_ of 368 nM, MTT assay; *p* < 0.05) and proliferation pattern (real-time cell analysis assay) was shown after 48 h exposure with Geo A. The cell proliferation blockade was confirmed after 24 h of Geo A treatment at 500 nM, with a 46% (*p* < 0.0001) reduction in the total number of cells (cell counting) and G2/M phase cell cycle arrest. Other cytotoxic evidence such as DNA fragmentation, phosphatidylserine exposure (flow cytometry), and time-dependent plasma membrane damage (Trypan Blue) suggested cell death by apoptosis. Therefore, Geo A showed both cytostatic and cytotoxic effects on A431 cells. Taken together, these data point out Geo A as a promising therapeutic molecule for NMSC treatment and is the first depsipeptide (marine or terrestrial), to our knowledge, to target this type of cancer cell.

## 1. Introduction

The uncontrolled proliferation and spread of abnormal cells are hallmark features of cancer, a group of diseases responsible for more deaths worldwide than infectious and parasitic diseases (AIDS, tuberculosis, and malaria) combined [1]. In developing countries, cancer is the third leading cause of death, following cardiovascular, infectious, and parasitic diseases. In wealthy countries, the disease is the second cause of death, after cardiovascular diseases [1,2,3,4]. The number of new cancer cases in 2020 was 19.3 million worldwide, while the total cancer deaths were estimated in 10 million in the same year (27,397 deaths/day), according to GLOBOCAN data from the International Agency for Research on Cancer (IARC) [5].

Non-melanoma skin cancer (NMSC) has the highest incidence of all cancers in white populations, presenting two major forms, basal cell carcinoma (BCC) and squamous cell carcinoma (SCC), which are malignant epithelial neoplasms. NMSC is usually non-lethal and generally unrecognized as being of public health significance, although patients’ treatment costs during hospitalization are at least two times higher than for melanoma treatment in Germany [6]. NMSC ranks as the 5th most common cancer; an estimated 1,234,533 people globally were recently diagnosed with non-melanoma skin cancer, and approximately 69,416 died from this disease [7]. However, the World Health Organization (WHO) estimates 2–3 million cases per year of NMSC, which is likely underestimated since its communication to cancer registries is not mandatory [1,3,6]. The main therapeutic treatments comprise surgical removal, systemic chemotherapy, radiation, and photodynamic therapy in some cases [8,9,10]. Nevertheless, tumor recurrence, incidence of metastasis, chemoresistance, and adverse effects (e.g., alopecia, fatigue, weight loss, muscle spasm, dysgeusia, occurrence of inflammation, among others) have been reported, therefore challenging treatment success [11].

Marine organisms are a rich source of different molecules with a great variety of biological activities [12,13]. Some of these molecules are in clinical trials or already on the market [14,15], attesting the therapeutic potential of marine natural products. For the past three decades, the assessment of cytotoxicity using in vitro techniques was indeed the leading bioactivity searched in novel marine compounds [16,17] and showed good results on finding scaffold structures for cancer treatment [18,19].

The U.S. National Cancer Institute (NCI) has a library of approximately 230,000 extracts from plants, marine organisms, bacteria, and fungi as a part of the Program for Natural Product Discovery (NPNPD). Since 1986, approximately 25,000 marine invertebrates and marine algae have been collected [20,21,22]. Marine sponges (phylum Porifera) were the most studied group in the last fifty years, considering all the literature on marine natural products. However, since the mid-1990s, the proportion of new molecules described from Porifera species has diminished significantly, along with an increasing interest in other groups of marine organisms [23].

Amongst the molecules of marine origin with cytotoxic activity, depsipeptides constitute a structurally versatile group of natural and synthetic molecules distinguished by the substitution of traditional amide bonds with ester linkages within their peptide backbone. This modification, together with the frequent presence of uncommon amino acids and macrocyclic frameworks, imparts notable conformational diversity and potent biological activity. Marine-derived depsipeptides, in particular, display strong anticancer, antiviral, and antimicrobial effects, often acting through cytoskeletal disruption or modulation of key signaling pathways. Their capacity to target malignant cells with high specificity, combined with activity against drug-resistant phenotypes, highlights their growing relevance as promising scaffolds for therapeutic development [24,25,26,27].

**Figure 1:**
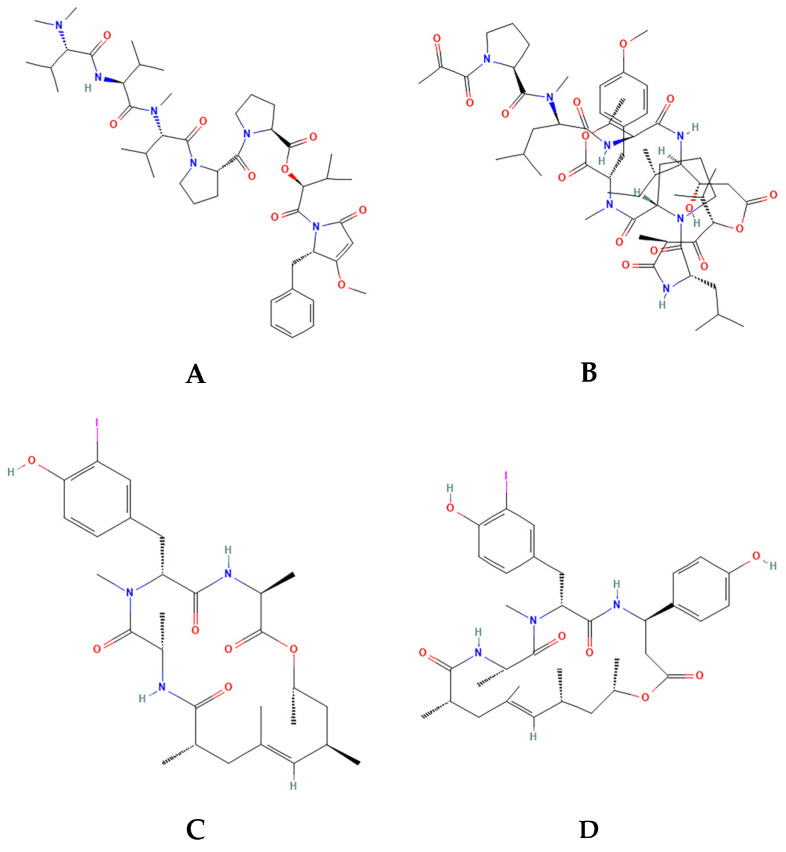
Two-dimensional structures of Dolastatin 15 (**A**), Dehydrodidemnin B (**B**), Geodiamolide A (**C**), and Geodiamolide H (**D**). Structures obtained from PubChem (https://pubchem.ncbi.nlm.nih.gov/).

Some marine-derived depsipeptides have reached clinical trials with indication against different types of cancer. Dolastatin 15 (Figure 1A), a seven-unit linear depsipeptide that contains four unique amino acids, dolavaline, dolaisoleucine, dolaproline, and dolaphenine, isolated from the sea hare *Dolabella auricularia* (phylum Mollusca) [28], is a potent antimitotic which affects microtubule polymerization and thus inhibits mitosis [29] and induces apoptosis [30]. Different synthetic analogs of dolastatin-15 were produced and underwent clinical trials as new chemotherapy drugs against melanoma, advanced solid tumors (colorectal, lung, kidneys, and pancreas), and others [31,32,33]. The depsipeptide dehydrodidemnin B (Figure 1B), also known as aplidine or plitidepsin, was synthesized by replacing the –OH group in the cyclic structure of didemnin B (*Aplidium albicans*) [34]. Plitidepsin demonstrated highly effective preclinical activity against severe acute respiratory syndrome coronavirus 2 (SARS-CoV-2), including in vivo efficacy in two mouse models of SARS-CoV-2 infection [35,36]. Regarding anticancer activity, plitidepsin blocks the cell cycle and induces apoptosis, thus having a strong antiproliferative effect against multiple myeloma cells (phase III of clinical trials) and for solid and hematological malignant neoplasias (phase II of clinical trials) [15,37,38,39,40,41]. The pleiotropic effects of plitidepsin on cancer cells is probably due to binding to eukaryotic translation eEF1A2, resulting in cell cycle arrest, growth inhibition, and induction of apoptosis [42].

Geodiamolides A (Figure 1C) and H (Figure 1D) are three-unit cyclic depsipeptides from marine sponges that disrupt microfilaments of sea urchin eggs and human breast cancer cell lines [43]. In the same study, these depsipeptides were not cytotoxic to non-tumor cell lines and did not affect the normal distribution pattern of the microfilaments of these cells. Geodiamolide A’s structure has two alanine residues and the rare amino acid 3-iodo-N-metiltyrosine [44], while Geodiamolide H has the same structure with a β-tyrosine replacing one alanine [45]. Therefore, substitution of only one amino acid is very important to the antiproliferative properties of these depsipeptides, according to the cell type. Although some structure–activity relationships have been described, the specific molecular targets involved in these effects have not yet been fully elucidated [43,45,46,47]. Geodiamolide H was further tested in breast cancer cells cultures in a three-dimensional environment, showing stronger effect on the aggressive Hs578T cell line, inhibiting both migration and invasion, and even reversing its malignant phenotype to polarized spheroid-like structures [46]. Recently, the total synthesis of Geo H was achieved, also including a set of Geo H synthetic analogs. Synthetic Geo H and several of the proposed analogs displayed cytotoxicity in HeLa cells and antiproliferative effects on human umbilical vein endothelial cells HUVEC (ATCC CRL-1730) and human chronic myeloid leukemia cells K-562 (DSM ACC 10) [48].

The expressive cytotoxicity to different cellular targets induced by Geo A and H in breast cancer cells suggests that these molecules might also act in other cancer types such as NMSC. To date, there are no recordings of clinical trials for depsipeptides against NMSC [49]. Of more than 68,000 cancer clinical studies in the above database, only 157 target NMSC detection and treatment.

Therefore, considering the high worldwide incidence and the need to improve therapeutic outcomes of NMSC, the goal of this work is to study the effects of the marine sponge depsipeptides Geodiamolide A and H in human non-melanoma skin cancer cells (A431, squamous cell carcinoma) in order to investigate their effects on cell proliferation and in cell death.

## 2. Results

**Figure 2:**
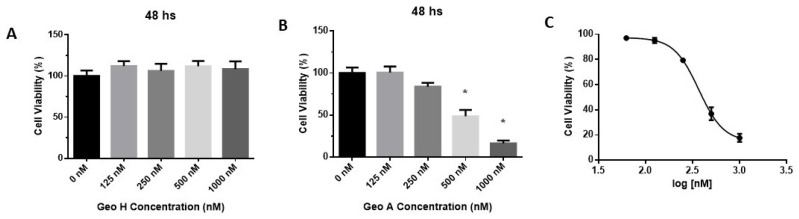
Cell viability of human squamous cell carcinoma cells (A431) after 48 h of incubation with Geodiamolide H (**A**) or Geodiamolide A (**B**); Paclitaxel (Ptx, 50 nM) was used as a positive control. Data on graph represent mean ± SEM (n = 6) and non-linear regression (log(inhibitor) (Geo A) vs. response—variable slope) (**C**). *: *p* < 0.05.

Geo A and Geo H showed a different cytotoxic pattern against A431 cells (Figure 2). While no significant statistical difference was observed after Geo H treatment, an expressive dose-dependent reduction in cell viability was shown after Geo A incubation (Figure 2A,B). After normalizing the raw data, it was observed that Geo A reduced the cell viability of A431 cells with IC_50_ of 368.4 nM and a 95% confidence interval from 313.1 to 433.4 nM (Figure 2C).

**Figure 3:**
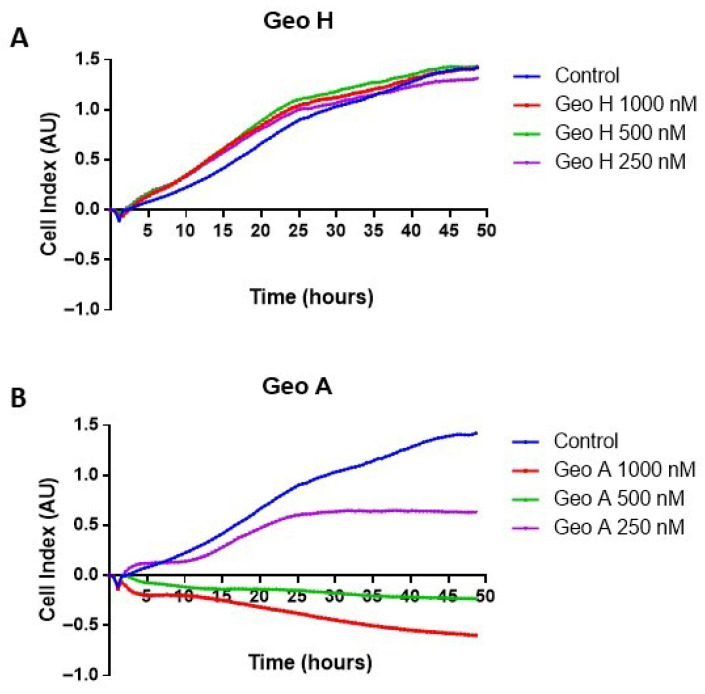
Cell proliferation pattern shown by RTCA using human squamous cell carcinoma cells (A431) in the presence of Geodiamolide H (**A**) or Geodiamolide A (**B**) in different concentrations (250 and 500 nM and 1 µM) during 48 h of incubation. Cells with culture medium were used as control group.

The reduction in the proportion of A431 viable cells, observed in the MTT assay after Geo A treatment, may indicate the occurrence of cell death induction and/or cell cycle blockade. In order to further investigate this cell viability reduction along the entire incubation time-lapse, RTCA experiments were conducted. An expected proliferation profile of A431 cells in the control wells (with culture medium only) was observed (Figure 3). The cells treated with Geo H showed no evidence of change in cell proliferation pattern at all concentrations evaluated (Figure 3A). On the other hand, Geo A induced a time- and dose-dependent effect on A431 growth (Figure 3B). At a Geo A concentration of 250 nM, the cells showed progressive growth in the first 20 h of incubation. After this period, proliferation ceased, and the cell index remained constant, indicating a cell proliferation blockade. At higher Geo A concentrations (500 nM and 1 µM) the depsipeptide induced a notable reduction in cell adherence in the first three hours, indicating cell death induction (Figure 3B). The early loss of adherence observed in RTCA was interpreted as an indirect indication of cell death based on impedance changes, and this inference was later corroborated by complementary assays, including Trypan Blue exclusion, Annexin V/PI staining, and DNA fragmentation.

**Figure 4:**
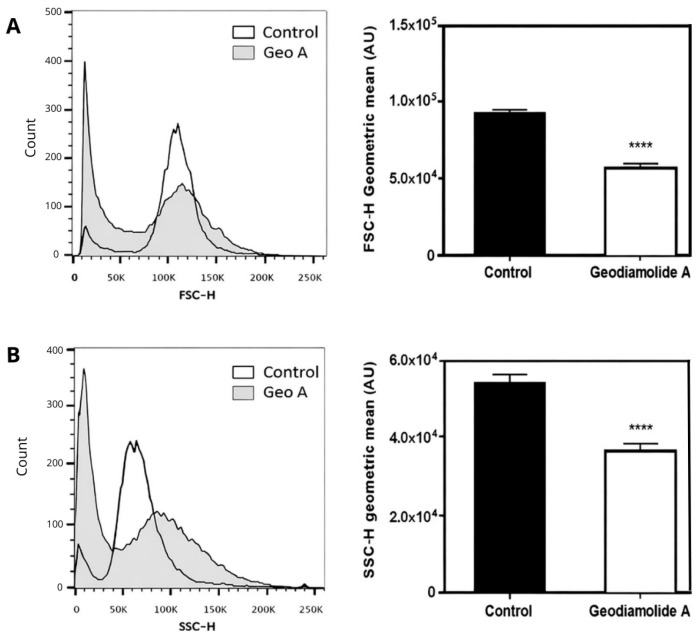
Cell size (FSC) (**A**) and intracellular granularity (SSC) (**B**) of human squamous cell carcinoma cells (A431) treated with Geodiamolide A at 500 nM for 48 h. ****: *p* < 0.0001.

The concentration of 500 nM of Geo A was used in further experiments to determine cell cycle alterations and cell death processes. The 500 nM concentration was chosen because it produced the most consistent early reduction in cell adherence, whereas 250 nM showed only modest effects and 1 µM produced a similar response to 500 nM. Cell morphology analysis from flow cytometry assays indicated that Geo A, after 48 h, reduced the size (FSC) of A431 cells by 38% (*p* < 0.0001) (Figure 4A) and also reduced the intracellular granularity/complexity (SSC) of cells by 35% (*p* < 0.0001) (Figure 4B) when comparing both geometric means with the control.

**Figure 5:**
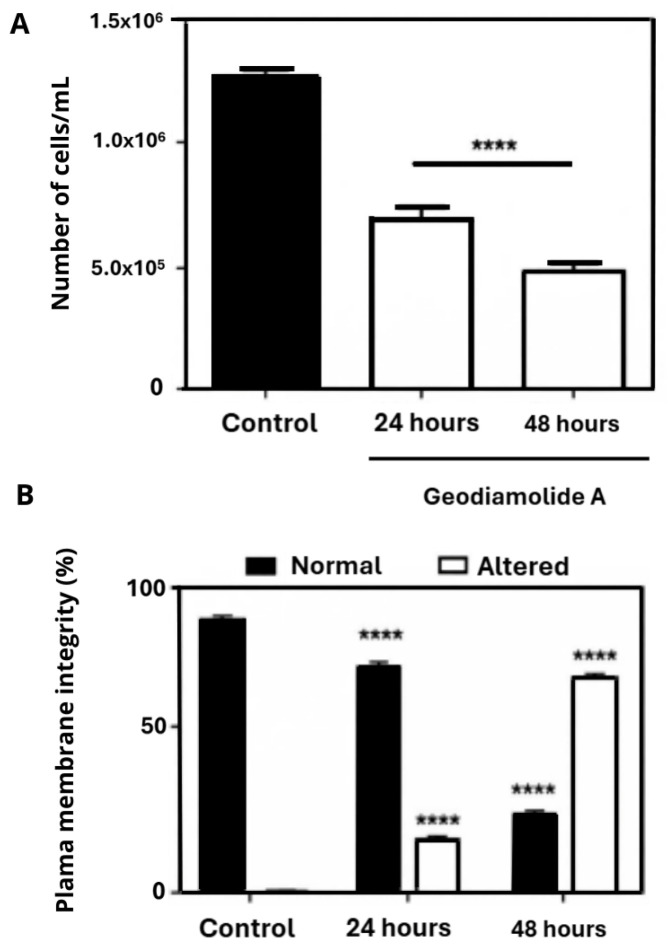
Total cell counting (**A**) and plasma membrane integrity (**B**) analyzed by Trypan Blue exclusion test of human squamous cell carcinoma cells (A431) treated with Geodiamolide A at 500 nM for 24 and 48 h. ****: *p* < 0.0001.

Results indicated that the depsipeptide Geo A promoted a significant (*p* < 0.0001) reduction in total cell number of 46 and 65% when compared to the control group after 24 and 48 h, respectively. Increases of 18 and 75% (*p* < 0.0001) in cells with altered membrane integrity were also observed after 24 and 48 h, respectively (Figure 5). The decrease in total cell counting and the increase in damaged plasma membranes are more expressive according to the exposure time.

**Figure 6:**
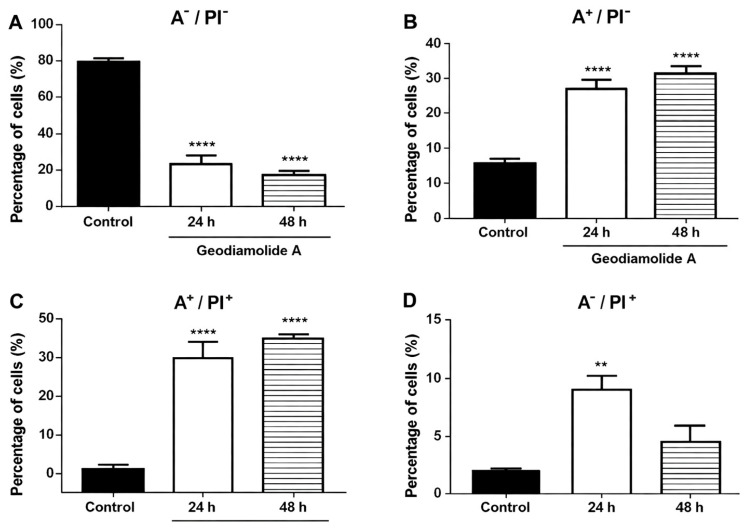
Cell death evaluation of human squamous cell carcinoma cells (A431) using Annexin V-FITC and Propidium Iodide (PI) after 24 and 48 h incubation with Geodiamolide A at 500 nM. Graphs represent: (**A**) viable cells or non-apoptotic (Annexin V^−^/PI^−^); (**B**) cells in early apoptosis (Annexin V^+^/PI^−^); (**C**) cells in late apoptosis or necrosis (Annexin V^+^/PI^+^); (**D**) cells in necrosis (Annexin V^−^/PI^+^). **: *p* < 0.01 and ****: *p* < 0.001.

Aspects of cell death were evaluated through phosphatidylserine exposure using Annexin V-FITC and PI to indicate apoptosis and membrane lesions, respectively. The results after 24 h of incubation showed a significant increase in the proportion of cells stained with Annexin V-FITC (34.7%, *p* < 0.001) and double staining—Anexin and PI—(31.7%, *p* < 0.001) when compared to control cells, indicating cells in apoptosis and also in late apoptosis stages (Figure 6).

After 48 h, it is possible to observe an increasing trend in the proportion of cells with Annexin V-FITC (39.4%, *p* < 0.001) and double staining (35.7%, *p* < 0.001) when compared to 24 h of incubation (Figure 6B,C). There is also decreasing trend in the proportion of viable cells (Figure 6A). These results suggest a time-dependent effect of Geodiamolide A on the A431 cell line.

**Figure 7:**
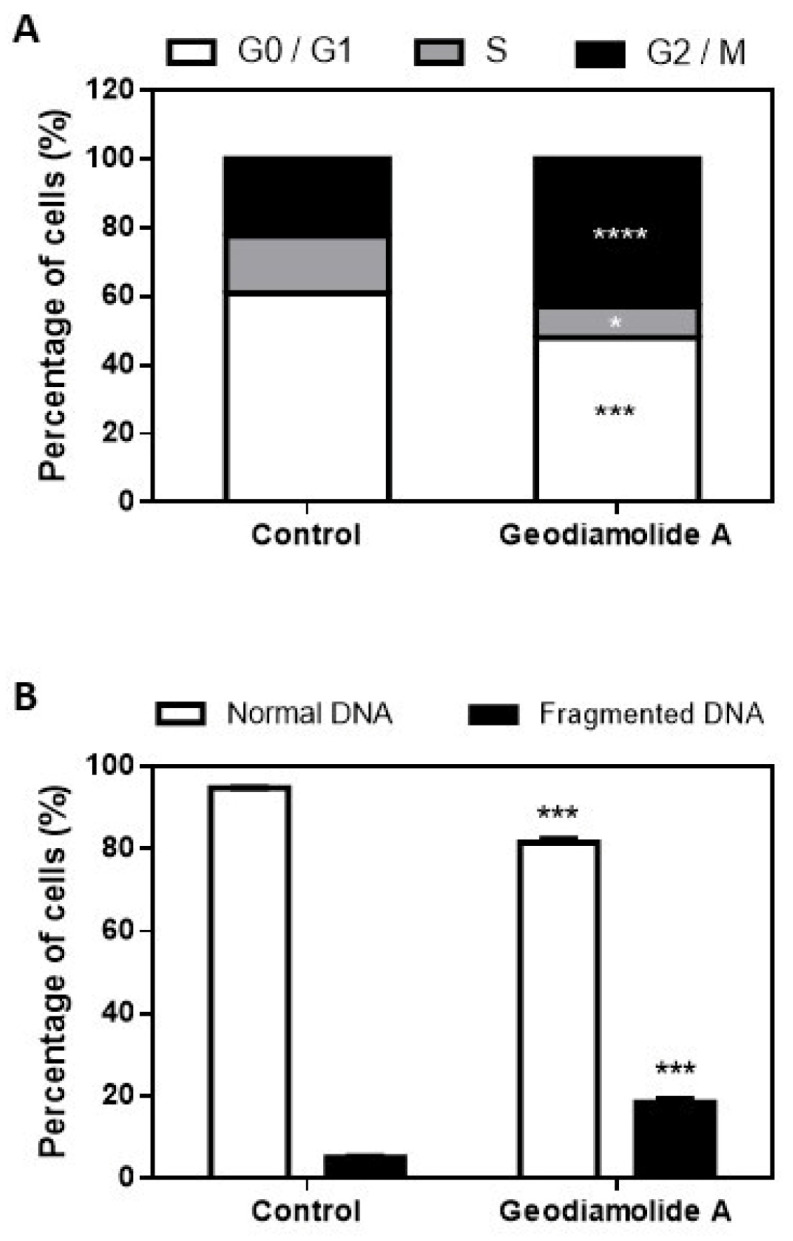
Cell cycle (**A**) and DNA fragmentation (**B**) of human squamous cell carcinoma cells (A431) treated with Geodiamolide A at 500 nM for 24 h. *: *p* < 0.01; ***: *p* < 0.001; ****: *p* < 0.0001.

DNA fragmentation and cell cycle of A431 treated cells were analyzed only for the time of 24 h of exposure since less expressive plasma membrane damage was observed in this time point in the Trypan Blue assay (Figure 5B). Flow cytometry experiments with PI staining showed that Geo A, after 24 h, increased the number of cells with fragmented DNA (20%, *p* < 0.0001) and the proportion of cells in G2/M phase (42.8%, *p* < 0.0001) and reduced the number of cells in G0/G1 and S phases (Figure 7).

## 3. Discussion

### 3.1. Geo A Effects on A431 Cells

In the present study, we focused on non-melanoma skin cancer cells (squamous cell carcinoma) instead of the previous studies with geodiamolides that focused on breast cancer cells. At first, the EC_50_ on the MTT assay was determined after 48 h of incubation (Figure 2). The MTT assay cannot discriminate between cytotoxic and antiproliferative effects [50]. MTT reduction is a marker reflecting viable cell metabolism and not specifically cell proliferation [51]. Geo H treatment did not affect A431 cell viability (Figure 2). Nevertheless, Geo A reduced A431 cells’ viability within a nanomolar range (IC_50_ of 368.4 nM), a concentration similar to the HTC cell lineage [47] but about 15 times higher when compared to breast cancer cells [43]. Jaspamide reduced leukemia cells’ (HL60) viability with an IC_50_ of ~100 nM [52], and at a higher concentration (~2 µM) against other transformed cell lineages such as Jurkat T cells, EL-4, SP-2/0 and J774.1 [53]. There are no studies testing jaspamide A against non-melanoma skin cancer cells [15].

Due to results of the cell viability, we further explored the dose and time dependence of cell proliferation through the real-time cell analysis (RTCA) assay (Figure 3). The control cells showed a crescent proliferation profile during the entire observation period (48 h). The proliferation profile of Geo H-treated cells remained similar to the control. In contrast, Geo A, at the lowest concentration tested (250 nM), allowed cell proliferation on the first day of incubation, causing the blockade of cell proliferation after 20 h. This effect can be due to actin depolimerization, which would prevent completion of the cell cycle and further proliferation. Similar results were observed when geodiamolides were incubated with sea urchin embryos in early stages of development [43]. At higher concentration, however, Geo A completely blocked cell proliferation, and due to the early reduction in cell adherence in the plaques after only three hours of incubation, cell death induction was suggested as a possible mechanism. Jaspamide also presented antiproliferative activity against several human prostate carcinoma cell lines (PC-3, LNCaP, and TSU-Pr1) due to actin disruption [54].

Here, a notable difference between Geo H and Geo A effects on both MTT and RTCA assays was observed. Structural variations among them are probably one of the keys of such opposite effects on A431 cells. Geo A has two alanine residues, while Geo H has one alanine substituted by a β-tyrosine. Moreover, these data strongly suggest that Geo A might interact with specific cell targets to trigger its cytotoxic effects on A431 cells.

The concentration of 500 nM was chosen for additional experiments with Geo A focusing on cell death evaluations because the depsipeptide was able to block cell proliferation and significantly reduce A431 cell viability.

Cell counting clearly indicated a reduction of 46 and 65% in the total cell number after 24 and 48 h of incubation with Geo A (Figure 5), respectively, corroborating the altered proliferation pattern shown by RTCA results. Reduction in cell proliferation may be due to the disruption of actin filaments caused by Geo A, as verified in previous studies [43,46,47], which might be related to the blockade of the cell cycle in the G2/M phase (Figure 7), preventing cytokinesis. Furthermore, it was possible to verify that cell membrane integrity was compromised (Figure 5). Cell membrane lesions are associated with two classical mechanisms of cell death: necrosis and the late stages of apoptosis [55]. These aspects were further investigated in the present study by additional flow cytometry assays (Figure 6 and Figure 7). DNA fragmentation, phosphatidylserine exposure staining by Annexin V-FITC, and DNA staining with PI (cell membrane damage) indicated the presence of cells in initial and late apoptosis stages after 24 and 48 h of incubation with Geo A. In addition, a reduction in cell size and intracellular granularity was also observed (Figure 4).

### 3.2. Comparative Context and Therapeutic Implications

Geodiamolide H, whose structure contains alanine, β-tyrosine, and a (R)-3-halo-N-metiltyrosine joined to a macrocyclic ring [45], is very similar to a well-studied marine sponge cyclic depsipeptide, jaspamide A. The structure of jaspamide A, isolated from the sponge *Jaspis johnstoni*, also contains three amino acid residues (L-alanine, β-tyrosine, and N-methyl-2-bromotryptophan) in a 15-carbon macrocyclic ring [56,57]. Geodiamolide H differs from jaspamide only by one amino acid residue (methyltyrosine instead of methyltryptophan) and its halogen substitute (the chemical structure of the first presents an iodine). Comparing jaspamide A to Geodiamolide A [44], the main differences in their structures are a second alanine residue in Geodiamolide A, instead of a β-tyrosine and a N-methyl-iodotyrosine instead of the N-methyl-2-bromotryptophan present in the jaspamide structure.

Jaspamide exerts its cytotoxic activity against cancer cells due to the stabilization of the microfilaments in vitro, but it can disrupt actin filaments in vivo [54,58], while the Geodiamolides A and H disorganize the actin filaments [43]. Further studies indicated that jaspamide induces cell death via apoptosis through caspase-3 activation, decreased Bcl-2 level, and increased Bax protein expression [59]. Cytoplasmic and membrane changes were also observed on apoptotic cells (myeloid leukemic HL-60 cell line) [52]. Interestingly, it has been suggested that other antitumor cyclodepsipeptides, such as homophymines, are not substrates for the efflux pump P-glycoprotein, which is mainly involved in tumor drug resistance [58,60]. Studies evaluating if Geo A can induce similar outcomes on tumor cells need to be further evaluated.

The present work described, for the first time, the antiproliferative and cytotoxic effects of Geo A on squamous cell carcinoma. Additional investigations in order to unravel the molecular targets, apoptosis pathway (intrinsic or extrinsic), participation of caspases, and the role of oxidative stress on Geo A mechanism of action will be the subject of future studies. The achievement of treatment success on cancer patients is challenging due to the heterogeneity and complexity of non-melanoma skin cancers [8,9,10,11]. Therefore, the antiproliferative and cytotoxic effects of Geo A described herein, together with previously reported evidence of low cytotoxicity toward non-tumor cells [43], make this molecule a promising antitumor candidate to be added to the current chemotherapeutic clinical arsenal.

## 4. Materials and Methods

### 4.1. Extraction and Isolation of Geodiamolides A and H

Geodiamolides A and H were extracted from specimens of *Geodia corticostylifera*, collected off the coast of São Paulo State, Brazil. The isolation of the peptides is described elsewhere [43]. Briefly, the specimens were homogenized in methanol, filtered, and partitioned in methanol–water (9:1, *v*/*v*)/n-hexane (1:2, *v*/*v*). Fractions were obtained by a step Pak Vac C_18_ cartridge (Millipore, Billerica, MA, USA) with stepwise elution of 20, 50, and 90% CH_3_CN in water. Finally, Geo A and Geo H were purified using CAPCELL PAK C_18_ (10 mm × 250 mm) (Shiseido Co., Ltd., Tokyo, Japan) under isocratic elution and analyzed by mass spectrometry (Thermo Scientific Orbitrap Elite, Waltham, MA, USA) to assess peptide quality.

### 4.2. A431 Cell Culture

The human non-melanoma skin cancer cell line A431, squamous cell carcinoma, was purchased from the Cell Bank of the Federal University of Rio de Janeiro (Rio de Janeiro, Brazil). The cells were routinely maintained in culture flasks (TPP, Trasadingen, Switzerland), at 37 °C in 5% CO_2_, in complete media containing Dulbecco’s Modified Eagle’s Medium (DMEM) with 100 IU/mL of penicillin and 100 μg/mL of streptomycin, supplemented with 10% (*v*/*v*) heat-inactivated fetal bovine serum (Life Technologies, Carlsbad, CA, USA).

### 4.3. Cell Treatment

Geodiamolides A and H were first dissolved at 1 mM in Milli-Q water and DMSO (9:1, *v*/*v*) and then diluted to 5 µM in DMEM. For cell viability assay (MTT), A431 cells were plated in 96-well culture plate at a density of 3 × 10^3^ cells/well and incubated overnight at 37 °C with 5% CO_2_. After medium replacement, cells were treated with varying concentrations of Geo A or Geo H (250 nM to 1 µM) prepared in complete medium. The concentration range was defined based on prior studies in breast cancer cell lines, since preliminary dose–response experiments in A431 cells were not conducted [43,46]. As a control group in all experiments, cells were treated with complete media (100% cell viability). A DMSO control was not necessary since the concentration in the tested samples was <0.05%, a level previously shown to have no effect on A431 cells [61]. Paclitaxel (50 nM) was used as a positive control. The plates were incubated for 48 h at 37 °C, 5% CO_2_ in a humid atmosphere.

For flow cytometry experiments, A431 cells were seeded in 12-well plates at 5 × 10^4^ cells per well and incubated for 24 h. The medium was then replaced with fresh medium containing 500 nM Geo A. After 24 and 48 h of treatment, cells were trypsinized (Life Technologies, Carlsbad, CA, USA), for 5 min, centrifuged at 560× *g* for 5 min, and prepared for subsequent assays.

Vehicle controls and untreated controls were included in all experiments under identical culture conditions. All consumables were obtained from single manufacturing batches to ensure methodological consistency.

### 4.4. MTT Assay

Cell viability was accessed using 3,4,5-dimethyl-thiazol-2,5 biphenyl tetrazolium bromide (MTT) (Life Technologies, Carlsbad, CA, USA) assay, which measures the reduction of tetrazolium salts by mitochondrial dehydrogenases in living cells to estimate metabolic activity, viability, and proliferation [62]. After 48 h treatment with Geo A and Geo H, 15 μL of MTT solution (5 mg/mL in PBS) were added to each well and incubated for 2 h at 37 °C with 5% CO_2_. The medium was then removed, 100 µL of DMSO were added, and absorbance was measured at 595 nm using a microplate spectrophotometer (SpectraMax, Molecular Devices, Sunnyvale, CA, USA).

### 4.5. Real-Time Cell Proliferation Analysis

Experiments were conducted using the xCELLigence RTCA DP system (Roche Diagnostics GmbH, Mannheim, Germany) housed in a humidified incubator at 37 °C and 5% CO_2_. Cell proliferation was monitored in 16-well E-plates (E-plate, Roche Diagnostics GmbH, Mannheim, Germany), where impedance readings were automatically converted to Cell Index values [63].

A431 cells (3 × 10^3^ per well) were seeded and allowed to attach for 30 min before plate insertion into the device. After 24 h, cells were treated for 48 h with Geo A or Geo H (250, 500 nM, or 1 µM) as described in Section 4.3, while control wells received complete medium. Each concentration was assessed in triplicate, and Cell Index values were recorded every 60 min over the 48-h period.

### 4.6. Plasma Membrane Integrity and Cell Count

Cell membrane integrity of A431 cells was measured by Trypan Blue assay as previously reported, in which unstained cells are considered intact and stained cells indicate membrane disruption [64]. Cells were treated with 500 nM Geo A for 24 and 48 h, then the medium was removed, cells were trypsinized, centrifuged, and resuspended in PBS 1X. A portion of the suspension (10 µL) was mixed to 20 µL of Trypan Blue solution (0.4% in phosphate buffer (Millipore Sigma, Burlington, MA, USA), and cells were counted in a Neubauer chamber (Millipore Sigma, Burlington, MA, USA) and classified based on the presence or absence of dye within the cytoplasm.

### 4.7. DNA Fragmentation Assay and Cell Cycle

Propidium iodide (PI) staining was used to assess DNA fragmentation and cell cycle distribution [65] in A431 cells. Treated cells were fixed in 70% cold ethanol and stored at −20 °C for 24 h, washed twice by PBS 1X, incubated with RNase (50 µM) for 30 min at 37 °C, protected from light, and then stained with PI (20 µg/mL, Probes, Thermo Fisher, Waltham, MA, USA) in PBS 1X for 30 min at room temperature, protected from light. before flow cytometric analysis (BD FACSVerse^TM^, Franklin Lakes, NJ, USA), with 10,000 events collected per sample.

### 4.8. Annexin-V FITC/Propidium Iodide (PI) Staining and A431 Morphologic Aspects

Apoptosis and necrosis were evaluated by Annexin V–FITC/PI staining after 24 and 48 h of Geo A treatment. In this assay, Annexin V–FITC detects phosphatidylserine externalization in apoptotic cells, whereas PI, which is impermeant to viable cells, identifies necrotic or membrane-compromised cells. Cells were washed in PBS 1X, resuspended in binding buffer [10 mM HEPES/NaOH (pH 7.4), 140 mM NaCl, 2.5 mM CaCl_2_], incubated with Annexin V–FITC (BD, Franklin Lakes, NJ, USA) and PI (50 µg/mL) for 15 min in the dark at room temperature, and analyzed by flow cytometry (BD FACSVerse™, Franklin Lakes, NJ, USA) counting 10,000 events/sample, also recording FSC and SSC parameters. Data were analyzed using Windows™ FloMax^®^ version 2.5 (Partec GmbH, Münster, Germany) and FlowJo version 7.6.3 (BD, Franklin Lakes, NJ, USA).

### 4.9. Statistical Analysis

The samples were tested in triplicate, and three independent experiments were performed. The results are represented as the means ± SEM. Significant differences were assessed by *t*-test or two-way analysis of variance (ANOVA) followed by Tukey’s post-test (α = 0.05) using the GraphPad Prism 6.01 software (GraphPad Software, San Diego, CA, USA).

## 5. Conclusions

Geodiamolide H did not show cytotoxicity to A431 cells. Conversely, Geodiamolide A effects on A431 cells can be divided into two major processes. First, the blockade of cell proliferation, evidenced by the reduction in cell proliferation (RTCA), the reduction in total number of cells (cell counting), and G2/M cell cycle blockade (Flow Cytometry). Second, cell death induction evidenced by pronounced effects such as DNA fragmentation, phosphatidylserine exposure (Flow Cytometry), and time-dependent plasma membrane damage (Trypan Blue)—all suggested to be related to early and late stages of apoptosis.

In conclusion, Geo A induces robust antiproliferative and cytotoxic effects in A431 cells, supported by complementary methodological approaches. Collectively, these findings position Geo A as a promising lead compound for the development of peptide-based therapeutic strategies against non-melanoma skin cancer.

## Data Availability

The original contributions presented in this study are included in the article. Further inquiries can be directed to the corresponding author.

## Abbreviations

The following abbreviations are used in this manuscript:

BCC	Basal cell carcinoma
Geo A	Geodiamolide A
Geo H	Geodiamolide H
IARC	International Agency for Research on Cancer
NMSC	Non-melanoma skin cancer
SCC	Squamous cell carcinoma
WHO	World Health Organization
